# Evaluation of knowledge, attitudes and vaccine hesitancy towards MMR vaccine among parents in the United Arab Emirates

**DOI:** 10.1371/journal.pone.0324629

**Published:** 2025-05-20

**Authors:** Kamel A. Samara, Hiba Jawdat Barqawi, Deema M. Alhayali, Samah Mohamed Kannas, Rim M. Elmorsy, Eman Abu-Gharbieh

**Affiliations:** 1 Department of Clinical Sciences, College of Medicine, University of Sharjah, United Arab Emirates; 2 Jefferson Abington Hospital, Abington, Pennsylvania, United States of America; 3 Research Institute of Medical and Health Sciences, University of Sharjah, Sharjah, United Arab Emirates; 4 School of Pharmacy, The University of Jordan, Amman, Jordan; Haute Autorite de sante, FRANCE

## Abstract

**Background:**

The Measles, Mumps, and Rubella (MMR) vaccine is highly effective, but vaccine hesitancy (VH) is a key barrier to achieving herd immunity. This study aims to assess the general attitudes and knowledge of UAE parents regarding measles and the MMR vaccine while identifying determinants of VH.

**Methodology:**

A cross-sectional study was done from 15th April 2024 and 5th June 2024. This study recruited 462 parents residing in the UAE using convenience sampling. Recruitment occurred through various social media platforms including WhatsApp, X, and Instagram, as well as in public by approaching parents in parks and malls. Information collected included demographic factors, measles knowledge, MMR attitudes and practices as well general vaccine attitudes using the Parental Attitudes toward Childhood Vaccines (PACV) and the WHO Vaccine Hesitancy Scale (VHS). Univariate, bivariate (chi-squared tests) and multivariate (logistic regression) analyses were conducted to identify significant factors contributing to vaccine hesitancy.

**Results:**

Of 462 participants, 87% were female, 41% were young adults, and 13.0% were healthcare workers. 15.6% of parents had no knowledge about measles and 20.0% had not heard of the MMR vaccine before. Healthcare professionals and specialist doctors were the most trusted sources of information regarding measles. Symptoms and transmission were generally well-recognized but there was a lack of knowledge regarding measles’ severity, epidemiology, and complications. 11.5% of parents believed the MMR vaccine can cause autism and 21.65% of parents (95% CI: 18.1–25.6%) were vaccine hesitant. Intention to vaccinate against measles was the strongest predictor of reduced vaccine hesitancy (AOR: 0.242, 95% CI: 0.143–0.410), followed by being middle-aged/older (AOR: 0.466, 95% CI: 0.281–0.772) and interest in learning more about measles (AOR: 0.394, 95% CI: 0.185–0.840).

**Conclusions:**

In the UAE, there are knowledge gaps regarding measles and concerns regarding the Measles, Mumps, and Rubella vaccine’s safety and efficacy. Parents who refuse MMR vaccination often demonstrate general vaccine hesitancy, necessitating prompt action to reestablish trust in the local vaccine programs and promote vaccination.

## Introduction

Infectious diseases continue to pose a significant threat to public health, causing widespread illness and mortality [1]. Measles, Mumps and Rubella (MMR) is a combined vaccine that protects against viral infections, some of which can lead to serious long-term sequelae, including unborn children [2]. Measles, a highly contagious and potentially fatal illness, has been a leading cause of death among children [2]. It typically presents with fever and rash, as well as the characteristic cough, coryza and/or conjunctivitis. While there is no treatment for the disease, patients are usually managed supportively and treated for any secondary complications that may arise, such as pneumonia, diarrhea, or encephalitis [3]. The advent of the MMR vaccine has dramatically reduced the incidence of measles, virtually eradicating it in regions with high vaccination coverage and significantly decreasing mortality and morbidity rates [3]. The vaccine has been found to be highly effective even and is usually given twice reaching effectiveness of 97%. Additionally, and with more than 100 million doses administered since 2000, MMR vaccines have been shown to be safe with an acceptable side-effect profile (fever in <15% of participants; transient rash in ~5%; serious adverse events are single cases per each million-dose administered) [4].

Globally, measles vaccination is estimated to have prevented more than 20 million deaths during 2000--2015. During this period, measles incidence decreased from 146 to 36 cases per million-population [2]. One of the greatest causes of ambiguity and uncertainty regarding the vaccine’s safety is due to the infamous 1998 retracted Lancet paper that speculated a link between the MMR vaccine and autism. Even with well-established strong evidence of the vaccine’s safety and the lack of any link with autism, parents are still hesitant to have their children receive the vaccine [5]. This now-debunked association between the MMR vaccine and autism is still considered one of the main challenges that has derailed MMR vaccination campaigns globally [1–8]. This issue specifically is one instance of what has broadly been recognized and studied in the literature as vaccine hesitancy.

Despite the WHO’s strategic plans guiding global and regional eradication efforts, MMR vaccination rates remain insufficient, raising concerns about achieving eradication and preventing outbreaks---even in high-coverage countries [9]. In the MENA region, vaccine coverage gaps persist. While the UAE has reported high MMR vaccination rates in 2022 (98% for the first dose and 91% for the second) [10], neighboring countries have shown significant disparities. According to UNICEF, Iraq, Jordan, Syria, and Yemen have some of the lowest coverage levels, with Yemen’s DTP3 coverage at 72% and Syria’s at just 49% in 2020 [11]. This can be explained by the MMR vaccine being an essential part of the UAE’s National Immunization Program, with all vaccination services being provided free of charge for children under five [12]. This ensures high coverage rates; however, achieving 100% coverage remains a challenge with a clear need for identifying and addressing the gaps through targeted interventions that enhance immunization efforts in the region.

The WHO SAGE Working Group on Vaccine Hesitancy defines vaccine hesitancy as a delay in acceptance or refusal of vaccination despite its availability [13]. Vaccines have consistently been recognized as one of the most cost-effective health technologies of all time, substantially reducing morbidity and mortality worldwide and revolutionizing preventive care [14]. However, millions of people globally do not benefit from vaccines, whether due to a lack of access, ability, or will [15]. Vaccine hesitancy (VH) has been identified as one of the main drivers behind the declining (or in some cases reversing) progress for vaccination campaigns, including measles. VH undermines efforts to achieve herd immunity and fully vaccinate the population, both crucial for eradicating infectious diseases. Worryingly, unvaccinated individuals can act as virus reservoirs, causing outbreaks and delaying control efforts [16].

Vaccine hesitancy has been understudied throughout the Gulf region and the Arab world at large; Algabbani et al. conducted a systematic review looking at VH in the Gulf and found that hesitancy does exist, but there is a lack of research on the topic and its determinants [17]. In the UAE, while there is a lack of data, it is expected that the attitude towards MMR vaccine is multifactorial and influenced by sociodemographic factors like educational level and socioeconomic status as well as vaccine-related factors such as parents’ trust in healthcare providers and previous experiences and beliefs regarding vaccines. Information sources are also instrumental in shaping and developing vaccination attitudes, with healthcare professionals being the most common and trusted source [18]. As such, this study aims to evaluate the UAE’s parents’ general attitudes and knowledge toward measles and its vaccine as well as examine MMR vaccine refusal patterns and their relationship to general vaccine hesitancy.

## Materials and methods

### Study population, sampling techniques, and data collection

This cross-sectional study was conducted in the UAE between April 15, 2024, and June 5, 2024. We employed convenience sampling to ensure a diverse representation of parents from different backgrounds. Eligible participants included parents currently living in the UAE with at least one child. Recruitment occurred through multiple channels including social media platforms (WhatsApp, X, and Instagram) and in-person approaches at public spaces such as parks and malls. The required sample size was determined using Cochran’s formula, assuming a 95% confidence level, a 5% margin of error, and a standard error of 1.96, P = 0.5, (assumed proportion of vaccine hesitancy), and (margin of error). The calculated minimum sample size was 385. The final sample size of 462 responses was achieved after excluding those not meeting the inclusion criteria. Prior to participation, all respondents reviewed a Participant Information Sheet outlining the study’s purpose and procedures. Data quality assurance measures included pilot testing of the questionnaire to refine clarity and reliability, regular monitoring of collected responses to check for inconsistencies, and the use of automated validation rules in Google Forms to minimize input errors. Confidentiality was ensured by not collecting any identifying information, and all data was securely stored with access restricted to the research team.

### Data collection tools, procedures, and quality assurance

Data was collected using a structured online questionnaire hosted on Google Forms. This questionnaire, adapted from previously validated instruments, included a mix of multiple-choice, Likert-scale, and open-ended questions covering demographics, vaccine attitudes, and practices. Prior to the full-scale study, the questionnaire underwent pilot testing to assess clarity and reliability, ensuring it was culturally appropriate and easily understood by the target population. To ensure standardized and accurate data entry, automated validation rules were implemented within Google Forms. These rules minimized input errors and ensured that responses met the required format. Additionally, regular monitoring of incoming data was performed to identify and address any inconsistencies or incomplete submissions promptly. Together, these measures contributed to robust data quality assurance, ensuring that the final dataset was both reliable and suitable for subsequent statistical analysis.

### Questionnaire development

The questionnaire was adapted from previously validated tools used in similar studies [19–21]. It incorporated the Parental Attitudes toward Childhood Vaccines (PACV) scale [22] and the World Health Organization’s Vaccine Hesitancy Scale (VHS) [23]. The final 67-item questionnaire comprised four main sections: demographics, measles knowledge, MMR vaccine attitudes and practices, and general vaccine attitudes. We included a combination of yes/no questions, single- and multi-select items, and Likert scale-based questions to comprehensively assess participants’ perspectives. Initially developed in English, the questionnaire was translated into Arabic to ensure accessibility. We utilized the validated Arabic version of the PACV scale [24].Following pilot testing, feedback was incorporated as appropriate. The finalized questionnaire was hosted on Google Forms for ease of distribution. The study protocol and questionnaire received ethical approval from the University of Sharjah Research Ethics Committee (Reference Number: REC-24-03-04-01-F) and adhered to all relevant ethical guidelines and regulations. No identifying information was collected from participants.

### Study variables

The analysis included the following key variables:

#### Independent variables.

- Demographics: Age, gender, nationality, education level, employment status, and income.
- Vaccine knowledge and awareness: Understanding of measles, MMR vaccine, and general vaccine-related information.
- Attitudes toward vaccines: Measured using responses to specific statements regarding vaccine hesitancy.
- Intention to vaccinate youngest child against measles: Measured by a direct question asking if parents plan to vaccinate their youngest child against measles.

#### Dependent variable.

- Vaccine hesitancy: Categorized based on responses to vaccine-related questions using the PACV scale, with a score ≥50% indicating vaccine hesitancy.
- MMR vaccine refusal/hesitancy: While no validated tool specifically for MMR hesitancy was used, we examined responses to questions about intention to vaccinate the youngest child against measles and beliefs about MMR vaccine safety, including perceived association with autism.
- For certain variables with limited responses in some categories (such as vaccination status of children), response categories were combined (e.g., “some” and “all” children vaccinated) to ensure sufficient statistical power for analysis.

### Data analysis

Data processing and analysis were performed using Python (version 3), specifically employing the Matplotlib (v3.3.4), pandas (v1.2.4), and statsmodels (v0.12.2) libraries, following methodologies similar to those adopted in previous papers such as Barqawi et al. (2024).

#### Descriptive analysis.

Baseline and demographic characteristics were summarized using descriptive statistics. Continuous variables were reported as means (with standard deviations) or medians (with interquartile ranges), while categorical variables were presented as frequencies and percentages. Five-point Likert scale responses were collapsed into tertiary variables according to the scoring guidelines for the Parental Attitudes toward Childhood Vaccines (PACV) and the World Health Organization’s Vaccine Hesitancy Scale (VHS). Specifically, responses indicating agreement or strong agreement with vaccine-hesitant statements were categorized as “hesitant,” those indicating disagreement or strong disagreement were classified as “non-hesitant,” and neutral or “I do not know” responses were coded as “unsure”.

#### Analytical analysis.

Bivariate analyses were conducted using chi-squared tests to examine associations between vaccine hesitancy and independent variables. To control for potential confounding factors and identify independent predictors of vaccine hesitancy, a multiple logistic regression analysis was performed. This model calculated Adjusted Odds Ratios (AOR) with 95% confidence intervals (CI) for each predictor. Variables that demonstrated significant associations (p < 0.05) in the bivariate analyses were included in the multiple logistic regression model. Missing values were managed using pairwise deletion, and a p-value of less than 0.05 was considered statistically significant for all analyses.

## Results

### Demographics

Table 1 represents the full distribution of demographics for the 462 responses that were included. 87.01% (n = /402/462) were female, 41.01% (n = 187/456) were young adults (ages 18–39 years), 71.21% (n = 329/462) had a diploma or bachelor’s degree, 94.16% (n = 435/462) were married, 87.01% (n = 402/462) were non-Emirati Arab (other Arab), and 60.61% (n = 280/462) lived in Sharjah and other Northern Emirates. Healthcare workers were 12.99% (n = 60/462) of the sample, with 41.34% (n = 191/462) being housewives and 35.06% (n = 162/462) working non-healthcare jobs. The number of children in the household was highly variable, with only 13.42% (n = 62/462) having only one child. Similarly, the ages of the children also varied but more than half of the participants had at least one child aged 12–18 years. Only 67.75% (n = 313/462) of households had fully insured their children and 43.17% (n = 199/461) did not regularly visit a doctor for their children’s health.

**Table 1:** Demographics and baseline characteristics of participants.

Sex - % (n)		Do your children have health insurance? % (n)	
Female	87.01% (n = 402/462)	No, none of them	19.91% (n = 92/462)
Male	12.99% (n = 60/462)	Yes, but only some of them	12.34% (n = 57/462)
**Age - % (n)**		Yes, all of them	67.75% (n = 313/462)
Young adult (18–39 years)	41.01% (n = 187/456)	**Did you receive the COVID-19 vaccine? % (n)**	
Middle-aged/ old-aged adult (40 + years)	58.99% (n = 269/456)	Not received	6.93% (n = 32/462)
		1 dose/ 2 doses	48.48% (n = 224/462)
**Highest degree obtained - % (n)**		3 doses or more	44.59% (n = 206/462)
High school or lower	17.32% (n = 80/462)	**Did you receive the influenza vaccine last year? % (n)**	
Diploma/bachelor’s degree	71.21% (n = 329/462)	Yes	31.39% (n = 145/462)
Postgraduate degree (MSc, PhD., etc.) or higher	11.47% (n = 53/462)	No	68.61% (n = 317/462)
**Marital status - % (n)**		**Did children receive mandated MoH vaccines % (n)**	
Married	94.16% (n = 435/462)	Yes, all children	91.56% (n = 423/462)
Widowed/ divorced	5.84% (n = 27/462)	Yes, some of the children	2.60% (n = 12/462)
**Nationality - % (n)**		No, none/ not sure	5.84% (n = 27/462)
Non-Arab	2.38% (n = 11/462)	**Did children receive other non-mandated MoH vaccines % (n)**	
Other Arab	87.01% (n = 402/462)		
UAE national	10.61% (n = 49/462)	Yes, all children	46.54% (n = 215/462)
**Place of Residence - % (n)**		Yes, some of the children	11.26% (n = 52/462)
Abu Dhabi	24.03% (n = 111/462)	No, none/ not sure	42.21% (n = 195/462)
Dubai	15.37% (n = 71/462)	**How regularly do you visit your children’s doctor for checkups? % (n)**	
Sharjah and other northern emirates	60.61% (n = 280/462)		
**Field of work - % (n)**		I do not regularly visit	43.17% (n = 199/461)
Non-healthcare worker	35.06% (n = 162/462)	Every month	6.29% (n = 29/461)
Healthcare (doctor, nurse, dentist, pharmacist, etc.)	12.99% (n = 60/462)	Every 3 months	12.15% (n = 56/461)
Housewife	41.34% (n = 191/462)	Every 6 months	16.27% (n = 75/461)
Other (Unemployed; student; etc.)	10.61% (n = 49/462)	Annually	22.13% (n = 102/461)
**Number of children in household - % (n)**		Other	16.27% (n = 75/461)
1	13.42% (n = 62/462)	**How has the COVID-19 pandemic affected your opinions regarding vaccines? - % (n)**	
2	30.74% (n = 142/462)	More negative	29.87% (n = 138/462)
3	26.62% (n = 123/462)	No effect	45.24% (n = 209/462)
4 or more	29.22% (n = 135/462)	More positive	24.89% (n = 115/462)
**Age of children in household - % (n)**		**Which of the following would you use to learn more about measles? - % (n)**	
1 months - 11 months	7.58% (n = 35/462)	General practitioner/primary care pediatrician	42.3% (n = 195/461)
1 years - 2 years	11.69% (n = 54/462)	Specialist doctors	32.54% (n = 150/461)
3 years - 5 years	23.59% (n = 109/462)	Governmental websites (CDC, MOHAP, DHA, DOH, WHO, etc.)	29.50% (n = 136/461)
6 years - 11 years	35.93% (n = 166/462)	Social media	27.11% (n = 125/461)
12 years - 18 years	52.60% (n = 243/462)	Mass media (radio/tv/newspapers)	12.15% (n = 56/461)
19 years or older	33.77% (n = 156/462)	I do not use any sources	16.05% (n = 74/461)

While 91.56% (n = 423/462) of parents reported all their children having received all mandated vaccines, only 46.54% (n = 215/462) reported the same when it came to non-mandated vaccines. As for the parents’ own vaccination practices, 44.59% (n = 206/462) of parents had received 3 doses or more of the COVID-19 vaccine and 31.39% (n = 145/462) had received the influenza vaccine. Only 24.89% (n = 115/462) reported more positive attitudes about vaccination due to the COVID-19 pandemic, with 45.24% (n = 209/462) reporting no change and 29.87% (n = 138/462) reporting more negative ones. Finally, participants were asked which information source they would use to learn more about measles: general practitioners and primary care pediatricians were the most common (42.3%, n = 195/461) followed by specialist doctors (32.54%, n = 150/461), governmental websites (29.50%, n = 136/461) and social media (27.11%, n = 125/461).

### Measles knowledge

Of the 462 parents, 12.34% (n = 57) reported at least one of their children having contracted measles. 15.58% (n = 72/462) of participants stated that they are not at all knowledgeable regarding the measles virus; as such, only 390 of the participants filled the measles knowledge section with Table 2 detailing their responses. 56.41% (n = 220/390) of parents were aware that measles does not only affect children. Transmission was well-understood with 76.92% (n = 300/390) recognizing that the virus is transmitted through direct/indirect contact. However, only 56.15% (n = 219/390) were aware of how infectious measles can be. Several symptoms were also well-recognized, specifically fever (74.62%, n = 291/390) and rash (70.51%, n = 275/390). Complications on the other hand were not well-known: 44.36% (n = 173/390) chose “I do not know” making it the most common response to that question. Similarly, most parents were unclear regarding measles’ severity and burden. Even more, only 15.38% (n = 60/390) correctly recognized that there is no treatment for measles. Overall, nearly two-thirds of the sample were not very concerned about their children contracting measles or were unsure of the risk.

**Table 2:** Parent’s measles knowledge.

Have any of your children ever had measles? % (n)		How knowledgeable are you about measles? % (n)				
No	79.22% (n = 366/462)	Not at all knowledgeable			15.58% (n = 72/462)	
Yes	12.34% (n = 57/462)	Somewhat knowledgeable			48.05% (n = 222/462)	
Not sure	8.44% (n = 39/462)	Knowledgeable/ very knowledgeable			36.36% (n = 168/462)	
**What do you think are some of the symptoms of measles? % (n)**		**What do you think are some of the possible complications of measles? % (n)**				
Fever	74.62% (n = 291/390)	Brain damage			27.44% (n = 107/390)	
Rash	70.51% (n = 275/390)	Lung infection			25.9% (n = 101/390)	
Feeling unwell	45.64% (n = 178/390)	Ear infections			25.38% (n = 99/390)	
Cough	35.64% (n = 139/390)	Heart problems			18.46% (n = 72/390)	
Headache	31.79% (n = 124/390)	Blindness			15.64% (n = 61/390)	
Red and watery eyes	27.18% (n = 106/390)	None of the above			4.87% (n = 19/390)	
Vomiting	13.08% (n = 51/390)	I don’t know			44.36% (n = 173/390)	
Belly pain	9.23% (n = 36/390)	**How dangerous do you think measles? % (n)**				
Neck stiffness	8.97% (n = 35/390)	Not dangerous			7.69% (n = 30/390)	
Constipation	4.87% (n = 19/390)	Somewhat dangerous			49.74% (n = 194/390)	
Measles has no symptoms	1.54% (n = 6/390)	Very dangerous			24.36% (n = 95/390)	
I don’t know	8.72% (n = 34/390)	Unsure			18.21% (n = 71/390)	
**For each of the following, select the most accurate statement**						
**Statement**		**Yes**	**No**			**I do not know**
**There is no treatment for measles.**		15.38%* (n = 60/390)	49.74% (n = 194/390)			34.87% (n = 136/390)
**An infected person with measles can infect up to 90% of unvaccinated people they come in contact with.**		56.15%* (n = 219/390)	11.28% (n = 44/390)			32.56% (n = 127/390)
**Measles can only affect children.**		13.59% (n = 53/390)	56.41%* (n = 220/390)			30.0% (n = 117/390)
**Needing hospitalization is rare if someone is infected with measles.**		30.77%* (n = 120/390)	33.08% (n = 129/390)			36.15% (n = 141/390)
**Up to 3 of every 1000 children infected with measles will die.**		28.97%* (n = 113/390)	7.44% (n = 29/390)			63.59% (n = 248/390)
**1 out of every 1000 children infected with measles will develop intellectual disability or hearing loss.**		29.49%* (n = 115/390)	8.46% (n = 33/390)			62.05% (n = 242/390)
**How many cases of measles do you think are recorded in the UAE every year? % (n)**						
Only a few cases				11.28% (n = 44/390)		
Tens of cases				9.23% (n = 36/390)		
Hundreds of cases and more				11.54% (n = 45/390)		
Unsure				67.95% (n = 265/390)		
**How concerned are you that you or a loved one might get measles? % (n)**		**How interested would you be in learning more about measles? %(n)**				
Not concerned at all/not too concerned	46.67% (n = 182/390)	Not interested/somewhat interested			41.43% (n = 191/461)	
Not sure	16.67% (n = 65/390)	Neutral			31.45% (n = 145/461)	
Somewhat concerned/ very concerned	36.67% (n = 143/390)	Interested/very interested			27.11% (n = 125/461)	

### Vaccination attitudes and practices

#### MMR vaccine-specific attitudes and practices.

Parents were asked about their practices and views regarding the MMR vaccine, results of which are presented in Table 3. 19.96% (n = 92/461) had never heard of the MMR vaccine before; doctors and healthcare professionals were the most common source the participants had heard about the vaccine from (45.12%, n = 208/461). When it came to MMR vaccine practices, 77.49% (n = 358/462) of parents had some or all their children receive the MMR vaccine, with 16.02% (n = 74/462) unsure about the vaccination status. Similarly, 76.19% (n = 352/462) were intending to vaccinate their youngest against measles. A fifth of participants did not know about possible side effects of the vaccine; fever was the most expected side effect at 58.87% (n = 272/462) followed by rash (36.36%, n = 168/462), and measles (18.83%, n = 87/462). 11.47% (n = 53/462) of parents believed that autism is a possible side effect of the vaccine. When exploring MMR vaccine refusal, responses varied greatly with no clear driver. Of the 103 participants who gave a reason, not knowing about the vaccine was the most common (34.95%, n = 36/103) followed by a lack of recommendation (14.56%, n = 15/103), not being sure if the vaccine is needed (10.68%, n = 11/103), and worrying about side-effects (8.74%, n = 9/103).

**Table 3:** Parents’ MMR vaccination attitudes and concerns.

Where did you hear about the MMR vaccine? % (n)		Have your children received the MMR vaccine? % (n)	
I have not heard of the vaccine before	19.96% (n = 92/461)	Yes, some/all of them	77.49% (n = 358/462)
		Not sure	16.02% (n = 74/462)
Doctor or healthcare professional	45.12% (n = 208/461)	No, none of them	6.49% (n = 30/462)
		**Do you plan to vaccinate your youngest child against measles? % (n)**	
Family or friends	11.28% (n = 52/461)	No	6.28% (n = 29/462)
Social media	8.89% (n = 41/461)	Unsure	17.53% (n = 81/462)
Other	14.75% (n = 68/461)	Yes	76.19% (n = 352/462)
**Which of the following you think is/are possible side effects of the MMR vaccine? % (n)**			
Fever			58.87% (n = 272/462)
Rash			36.36% (n = 168/462)
Measles			18.83% (n = 87/462)
Autism			11.47% (n = 53/462)
Blindness			10.61% (n = 49/462)
Other side-effects			10.61% (n = 49/462)
There are no side-effects			3.25% (n = 15/462)
I do not know			22.73% (n = 105/462)
**Which of the following are reasons you did not vaccinate your child/children against measles? % (n)**			
I vaccinated all my children			77.71% (n = 359/462)
Did not know about vaccine			7.79% (n = 36/462)
Doctor did not recommend			3.25% (n = 15/462)
Not sure if vaccine was needed			2.38% (n = 11/462)
I was worried about the side effects			1.95% (n = 9/462)
Doctor did not provide enough information			1.73% (n = 8/462)
Concerns about cost/insurance coverage			1.52% (n = 7/462)
Child had allergy or other health condition			1.52% (n = 7/462)
Did not think vaccine would work			0.87% (n = 4/462)
Influence by family/friends led me to decide against vaccine			0.87% (n = 4/462)
Child was afraid of needles/shots			0.65% (n = 3/462)
Religious beliefs			0.43% (n = 2/462)
Other			7.14% (n = 33/462)

#### General vaccine hesitancy.

**Fig 1:**
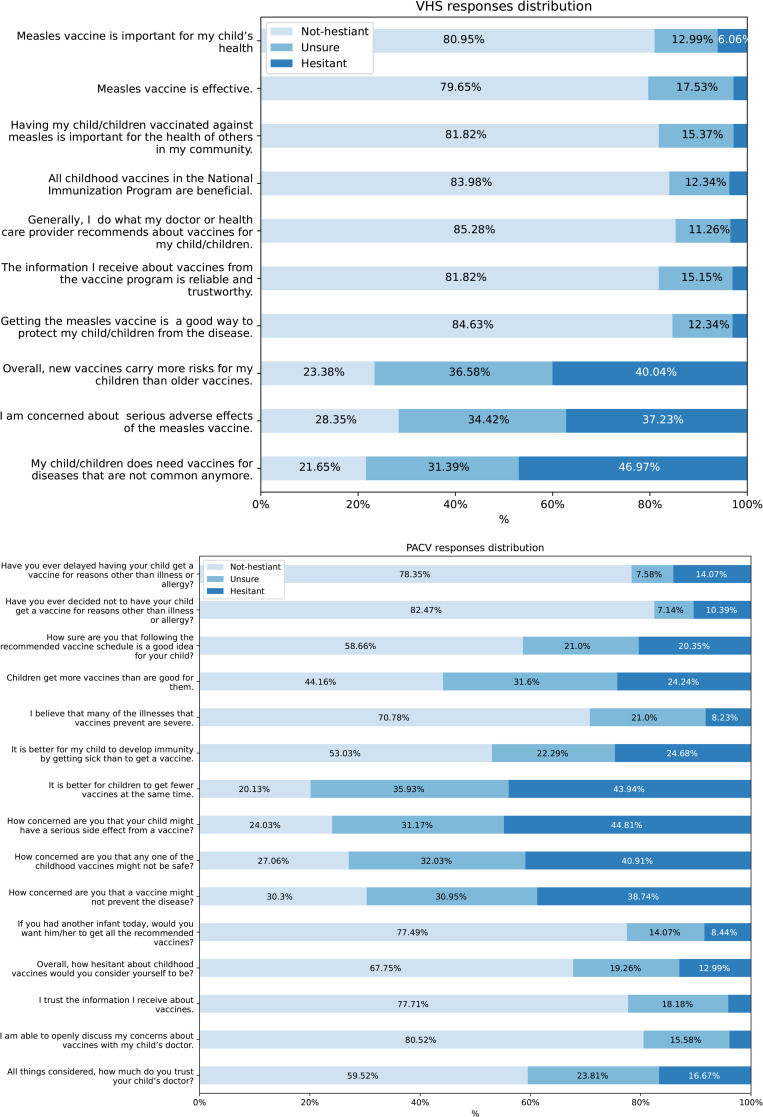
The distribution of responses for. (a) the measles-adapted Vaccine Hesitancy Scale (VHS) and (b) the general parental attitudes towards childhood vaccines.

Both the PACV (Cronbach’s α = 0.78, 95% CI: 0.75–0.81) and VHS (Cronbach’s α = 0.71, 95% CI: 0.67–0.75) showed acceptable internal consistency. For the VHS, attitudes were majorly non-hesitant except for items #8, #9, and #10 (as can be seen in Fig 1a). These three items were regarding risk perception as well as concerns regarding the need for vaccines for uncommon illnesses and serious adverse effects. Vaccine hesitant attitudes increased nearly eight-fold for these questions. The PACV scale showed more variability and distribution of responses (as can be seen in Fig 1b). Like the VHS, the biggest concerns had to do with vaccine’s efficacy and side effects with more than three-quarters showing hesitant or unsure attitudes. Parents were also asked about their reasoning behind delaying or refusing vaccines (shown in Table 4). When it came to delaying vaccines, forgetfulness was the most common (25.81%, n = 32/124) followed by lack of doctor recommendation (17.74%, n = 22/124), and fear of vaccine administration (16.13%, n = 20/124). Concern about side effects accounted for only 14.52% (n = 18/462) of vaccine delays. However, when it came to vaccine refusal, side effects predominated, being responsible for 29.57% (n = 34/115) of general vaccine refusals. Lack of recommendation by a doctor was second (21.74%, n = 25/115) and forgetfulness dropped to third, being reported by 14.78% (n = 17/115) of parents.

**Table 4:** Reasons for delaying or foregoing a vaccination; questions were part of the PACV questionnaire.

PACV #1 - What is the reason for delaying having your child get a vaccine? % (n)	
I did not delay any vaccines for reasons other than allergy or infection	73.16% (n = 338/462)
Forgetfulness	6.93% (n = 32/462)
Lack of recommendation by the doctor	4.76% (n = 22/462)
Fear of vaccine administration for your child	4.33% (n = 20/462)
Concerned about the side effects	3.90% (n = 18/462)
Vaccine was not available in the vaccination center	2.81% (n = 13/462)
Other	9.96% (n = 46/462)
**PACV #2 - What is the reason for not having your child get the vaccine? % (n)**	
I did not refuse any vaccines for reasons other than allergy or infection	75.11% (n = 347/462)
Concerned about the side effects	7.36% (n = 34/462)
Lack of recommendation by the doctor	5.41% (n = 25/462)
Forgetfulness	3.68% (n = 17/462)
Vaccine was not available in the vaccination center	3.03% (n = 14/462)
Having an objection to the administration of vaccines	2.81% (n = 13/462)
Other	7.58% (n = 35/462)

The PACV was used for establishing hesitancy. PACV scores had a mean (μ) = 32.12% and standard deviation (σ) = 18.06%; overall, 21.65% (n = 100/462) of participants were found to be hesitant (having met the traditional 50% cut-off for hesitancy). Bivariate analysis was conducted to determine which factors predicted vaccine hesitancy. The variables tested were the demographics (sex, age, educational level, marital status, nationality, place of residence, and field of work), intention to vaccinate the youngest child against measles, parent vaccination practices, previous measles experience, perceived knowledge regarding measles, frequency of doctor checkup, interest in learning more and the various information sources that can be used. Analysis of the relationship between health insurance status and vaccine hesitancy showed no statistically significant association (p = 0.058), though parents whose children lacked insurance trended toward higher hesitancy. Intention to vaccinate the youngest child against measles (p < 0.0005), age (p-value = 0.005), marital status (p-value = 0.006), perceived measles knowledge (p-value = 0.002), interest in learning more (p-value<0.0005), governmental websites as knowledge sources (p-value = 0.006), specialist doctors as knowledge source (p-value = 0.009), and having received the influenza vaccine last year (p-value = 0.010) were all found to be significant at the bivariate level.

The multivariate logistic regression model (Table 5) identified intention to vaccinate the youngest child against measles as the strongest predictor of overall vaccine hesitancy (AOR: 0.242, 95% CI: 0.143–0.410), with parents intending to vaccinate being significantly less likely to be vaccine hesitant. Being middle-aged/old-aged (AOR: 0.466, 95% CI: 0.281–0.772) and being neutral (AOR: 0.508, 95% CI: 0.273–0.946) or interested (AOR: 0.394, 95% CI: 0.185–0.840) in learning more about measles were also associated with lower overall vaccine hesitancy. Notably, while marital status was significant in bivariate analysis, it did not remain significant in the multivariate model (p = 0.129).

**Table 5:** The results of the logistic regression modeling general vaccine hesitancy and its determinants.

General Vaccine Hesitancy Predictors – Binary Logistic Regression (LR)						
Model Terms		$\left(e^{\beta_{i}}\right)$	95% CI for OR	SE	z-Statistic	P value
**Intercept (**$\beta_{0}$)		**4.190**	**1.408 - 12.466**	**0.556**	**2.575**	**0.010**
**Intention to vaccinate youngest against measles (P value: < 0.0005)**	No	–	–	–	–	–
	Yes	**0.242**	**0.143 - 0.410**	**0.269**	**-5.280**	**<0.0005**
**Age (P value: 0.005)**	Young adult	–	–	–	–	–
	**Middle-aged/ old-aged adult**	**0.466**	**0.281 - 0.772**	**0.258**	**-2.965**	**0.003**
**Marital status (P value: 0.006)**	Widowed/ divorced	–	–	–	–	–
	**Married**	0.501	0.205 - 1.221	0.455	-1.520	0.129
**Perceived measles knowledge (P value: 0.002)**	Not at all knowledgeable	–	–	–	–	–
	Knowledgeable/ very knowledgeable	0.660	0.308 - 1.418	0.390	-1.065	0.287
	Somewhat knowledgeable	0.751	0.390 - 1.449	0.335	-0.853	0.393
**Interested in learning more about measles (P value: < 0.0005)**	Not interested	–	–	–	–	–
	**Interested/ very interested**	**0.394**	**0.185 - 0.840**	**0.386**	**-2.411**	**0.016**
	**Neutral**	**0.508**	**0.273 - 0.946**	**0.317**	**-2.136**	**0.033**
**Knowledge source - Government websites (P value: 0.006)**	No	–	–	–	–	–
	Yes	0.796	0.414 - 1.528	0.333	-0.686	0.493
**Knowledge source - Specialist doctors (P value: 0.009)**	No	–	–	–	–	–
	Yes	0.804	0.435 - 1.487	0.314	-0.695	0.487
**Received influenza vaccine last year (P value: 0.010)**	No	–	–	–	–	–
	Yes	1.245	0.734 - 2.113	0.269	0.814	0.416
**Log-Likelihood: -212.48**	**Log-Likelihood of Null Model: -237.06**	**Log-Likelihood Ratio P value: < 0.0005**				

P values for the bivariate Chi-square tests are below each variable below rows with significant p values are bolded. OR: odds ratio; CI: confidence interval; SE: standard error

### Discussion

This study collected responses from 462 parents evaluating their knowledge, attitudes, and practices regarding measles and its vaccine as well the general status of vaccine hesitancy in the United Arab Emirates. 15.58% of parents had no knowledge regarding measles, with HCPs being the most common source of information regarding illness. Symptoms and transmission were generally well-recognized by the participants, but multiple gaps were found especially regarding the disease’s severity, epidemiology, and complications. In terms of MMR vaccination, a fifth had never heard of the vaccine and a quarter of participants were unsure or would refuse to vaccinate their youngest child with the MMR vaccine. Nearly a tenth of participants believed that the vaccine is linked with autism. The most common reasons for not vaccinating against MMR included not knowing about the vaccine and not receiving a recommendation from a doctor, with fear of side effects ranking third (only 9 reported this as the main reason). In terms of general vaccination hesitancy, 21.65% of parents were found to be hesitant according to the PACV. Notably, intention to vaccinate the youngest child against measles emerged as the strongest predictor of lower vaccine hesitancy in our multivariate model, suggesting a strong connection between MMR-specific attitudes and general vaccine confidence. Other significant protective factors include being middle-aged/older and showing interest in learning more about measles.

### The rise and decline of MMR vaccination?

Vaccination, one of the greatest public health achievements, has been a victim of its own success; as disease transmission and prevalence plummets, the public tends to forget about the severity of the illness and possibly underestimate the risks of the disease and overestimate the risks of the vaccine [14]. Between 2000 and 2022, MMR vaccination has prevented 57 million deaths from measles; Nonetheless, in 2022, 136,000 deaths worldwide were due to the virus, with the majority of reported cases occurring in unvaccinated or under-vaccinated children [25]. Worryingly, global childhood immunization coverage dropped during the COVID-19 pandemic; only 83% of children received at least a single dose measles vaccine in 2023 (compared to 86% in 2019), with 74% overall also receiving a second dose. Vaccination levels this low are not enough to prevent measles outbreaks, and in fact, such outbreaks were reported in 103 countries over the last 5 years [26]. In fact, the WHO has documented 42,200 measles cases across Europe in 2023, up from 941 cases the previous year, representing approximately a 4400% increase in cases [27].

Problematically, vaccine hesitancy introduces pockets of susceptibility which measles, being a highly transmissible disease, exploits easily. This can and does lead to outbreaks which present serious financial and public health risk; large outbreaks in England during 2012 and 2013 were estimated to cost £4.4 million but could have been prevented through 11,793 vaccines (which would have had an estimated cost of £182,909–4% of the total measles outbreak cost) [28]. A US modeling study estimated that for even a 5% reduction in MMR coverage, a 3-fold increase in measles would be expected, costing the public sector an additional $2.1 million [29]. This issue is only expected to grow with the 2023 UNICEF State of the World’s Children report finding that childhood vaccine importance perception has declined more than 10 percentage points across Europe and Central Asia, with some countries seeing drops as high as 44% [30].

Parental decision-making regarding vaccines is complex, involving emotional, cultural, social, spiritual, and political factors [31]. In fact, Larson et al. showed that vaccine hesitancy (VH) determinants are extremely complex, “varying across time, place, and vaccines” [8,13]. Overall, VH appears to be a function of many determinants include contextual (communication and media, religion, social norms, etc.), organizational (accessibility and quality of vaccination services) and individual (attitudes, beliefs, and knowledge) determinants [6]. The 2023 UNICEF report outlined a confluence of factors that have helped vaccine hesitancy grow, specifically: the widespread uncertainty that surrounded the response to the pandemic, the proliferation and increasing access to misleading information, the declining trust in expertise and the political polarization of vaccines [30].

Moreover, only recently has VH become more well-recognized in the UAE and the Arab world at large, due to the COVID-19 pandemic. As such, results are still scarce, but some general patterns can be noticed. Alsuwaidi found 12% (n = 36/300) of their UAE sample to be vaccine hesitant (using the PACV scale) with 16% of the parents reporting delaying vaccines with 6% deciding to not vaccinate their children at all. Similar to this study, being divorced was found to be significant in determining vaccine hesitancy status. However, age, educational level and nationality were found to have no relation with hesitancy, even at the bivariate level [24]. Barqawi et al. conducted a similar study among parents post-COVID-19 and found that the proportion of vaccine hesitant patients had increased to 14% (n = 77/550), with physician knowledge source, higher digital vaccine literacy, and vaccine perception all being associated with lower vaccine hesitancy. In the study, the biggest contributors to hesitancy were the risk of side effects, the worry about newer vaccines, and the lack of a need for vaccines for uncommon illnesses anymore [32]. The results of their study aligned closely with those from this study; however, the significant determinants were different with information source not being a significant variable in this study. However, it is important to highlight that the proportion of hesitant parents seems to be increasing, reaching double what was originally reported by Alsuwaidi et al. back in 2020.

Another UAE study had looked at the population’s knowledge regarding measles in 2017 and found a lack of knowledge regarding the disease and its vaccine. Family and friends were the most common source of knowledge at 50.3% followed by the internet and social media at 35.4%. While 93.8% agreed that the measles vaccine is good, only 66.8% disagreed that the measles vaccine can cause autism (although the study did not objectively evaluate and report general vaccine hesitancy) [33]. Similar to this study, it seems that some aspects of measles knowledge tend to be well-understood in the UAE’s community (specifically symptoms and transmission). In contrast, this study highlights greater utilization of physicians as information sources as well as a smaller proportion believing the MMR vaccine causes autism. Barakat et al. also looked at the attitudes and hesitancy surrounding the measles vaccine in nearby Jordan. In their study, more than 85% of the participants displayed resistant or hesitant attitudes to the vaccine when asked directly. Upon conducting multivariate analysis, again the perceived safety and efficacy of the vaccine were statistically significant in predicting vaccine hesitancy (in addition to number of offspring and previous vaccination behaviors) [19]. More globally, an Australian MMR study found good recognition of measles symptoms but poor perception of risk, severity and complications (like that seen here). However, a much larger percentage of participants (61.2%) were unaware of the MMR vaccine side effects and only 8% of parents were found to refuse immunizing their children with MMR; these parents tended to be older and skeptical of the vaccine’s safety and efficacy as well as encountered someone who had suffered vaccine side-effects [20].

Overall, these results hint at a vaccine hesitancy paradox. On one hand, parents will state complete confidence in the vaccine, showing full awareness of how important and effective they can be. Yet, they will still express major concerns regarding vaccine side-effects and their necessity, albeit those concerns do not translate to quantifiable action, with the majority still choosing to vaccinate their children. This highlights the complexities of modeling hesitancy and points towards a more nuanced view of hesitancy that current tools and surveys may not be detecting (a sort of actionable and non-actionable vaccine hesitancy). For example, in the Jordanian study by Barakat, while more than 85% were hesitant or resistant to the MMR vaccine, more than 90% reported never declining or postponing vaccination in general; in fact, Jordan itself had seen great successes with its vaccination programs [19].

Interestingly, our finding that the intention to vaccinate against measles was the strongest predictor of general vaccine hesitancy suggests that attitudes toward the MMR vaccine may serve as a reliable indicator for broader vaccine confidence. This aligns with literature positioning measles vaccination as a cornerstone of public trust in immunization programs. Despite extensive publicity around unfounded concerns about the MMR vaccine, parents who accept this specific vaccine appear significantly more likely to accept vaccines in general. This highlights the potential value of focusing public health messaging on building confidence specifically in the MMR vaccine, as improvements here may positively impact overall vaccine acceptance. Contrary to some expectations, we found that health insurance status did not significantly predict vaccine hesitancy in our population, which may reflect the UAE’s policy of providing free vaccination services for young children, effectively removing financial barriers to essential immunizations.

### Recommendations and further actions

Tackling vaccine hesitancy requires broad actions, working on the individual, provider, and national level, including standardized and regular quantifying of vaccine hesitancy and its clusters, an independent and transparent vaccine safety system, and a communication strategy that helps recognize and alleviate parents’ concerns [34]. First, it is important to build up vaccine confidence by educating parents regarding the measles and the MMR vaccine’s safety and effectiveness [35]. Social media can play a massive role in this educational campaign. Kharaba et al conducted a UAE COVID-19 vaccine acceptance study among parents; they found that the positive information about the COVID-19 vaccine and its role in halting the virus on social media was significant in increasing parents’ confidence in the vaccine [36]. Moreover, there is a need for comprehensive and robust systems that can independently evaluate and validate the safety of vaccines post licensure such as the Vaccine Safety Datalink project or the Post-Licensure Rapid Immunization Safety Monitoring program [36]. Such systems can play an important role in reestablishing trust and transparency within the domestic vaccine programs.

Additionally, HCPs need to be more proactive in recommending vaccines to parents and providing strong science-based recommendations while respecting the patients’ autonomy. HCPs are and continue to be the most trustworthy source about vaccines for most of the public making their role vital. Sometimes however, scientific evidence alone is not enough to provide reassurance about vaccine safety [36]. The rise of patient-centered care has made many parents prefer to be active participants in their children’s health, necessitating that doctors use clear language and present the benefits and risks of vaccines clearly. It is also important to avoid using judgmental language and scare tactics. Motivational interviewing specifically has been shown to help HCPs address parents’ concerns more effectively [31]. Finally and more generally, it is essential to address some of the structural issues that may hinder vaccine uptake, such as vaccine accessibility and affordability [35].

### Limitations

Using a cross-sectional design does not allow for establishing causal or temporal relations such as between vaccine hesitancy and vaccination practices. Moreover, the sampling technique (convenience sampling) used introduces the risk of selection bias (with the sample of this study overrepresenting Arab females). Given the importance of vaccination, there is always the possibility of social desirability bias. Moreover, since the responses were not verified using medical records, there is a risk of recall and/or reporting bias. To minimize these limitations, we implemented robust data quality measures and statistical controls in our analysis. However, these potential biases may affect the generalizability of our findings to the broader UAE population. Finally and methodologically, it is also possible that conducting a qualitative study using semi-structured interviews with parents can prove valuable in understanding the reasons for vaccine hesitancy.

## Conclusion

In this study, UAE parents were found to be semi-knowledgeable regarding measles and overall supportive of the vaccine; still, significant major concerns existed regarding the vaccine safety and efficacy. Moreover, the results point towards increasing vaccine hesitancy in the population. MMR-specific vaccine hesitancy could be attributed to a lack of knowledge about the vaccine, a lack of recommendations, and insufficient information regarding the need for the MMR vaccine, in addition to concerns about its side effects. Parents’ education about measles and the MMR vaccine’s safety is essential; thus, educational campaigns are needed to increase awareness of the MMR vaccine’s efficacy, whether through social media or healthcare centers. Healthcare providers play a significant role in recommending the vaccine and addressing parents’ concerns.
